# Environmental Sources of Prion Transmission in Mule Deer

**DOI:** 10.3201/eid1006.040010

**Published:** 2004-06

**Authors:** Michael W. Miller, Elizabeth S. Williams, N. Thompson Hobbs, Lisa L. Wolfe

**Affiliations:** * Colorado Division of Wildlife, Fort Collins, Colorado, USA;; † University of Wyoming, Laramie, Wyoming, USA;; ‡ Colorado State University, Fort Collins, Colorado, USA

**Keywords:** chronic wasting disease (CWD), cervid, epidemiology, mule deer, *Odocoileus hemionus*, prion, transmissible spongiform encephalopathy (TSE)

## Abstract

Whether transmission of the chronic wasting disease (CWD) prion among cervids requires direct interaction with infected animals has been unclear. We report that CWD can be transmitted to susceptible animals indirectly, from environments contaminated by excreta or decomposed carcasses. Under experimental conditions, mule deer (*Odocoileus hemionus*) became infected in two of three paddocks containing naturally infected deer, in two of three paddocks where infected deer carcasses had decomposed in situ ≈1.8 years earlier, and in one of three paddocks where infected deer had last resided 2.2 years earlier. Indirect transmission and environmental persistence of infectious prions will complicate efforts to control CWD and perhaps other animal prion diseases.

Controlling and possibly eradicating animal prion diseases (*1*) are goals shared by the international community (*2**,**3*). However, progress toward eliminating prion diseases from food-producing animals worldwide has been hampered by incomplete knowledge about transmission and environmental persistence of these novel proteinaceous pathogens. Two prion diseases, scrapie of sheep and goats (*4**-**8*) and chronic wasting disease (CWD) of deer (*Odocoileus* spp.) and elk (*Cervus elaphus nelsoni*) (*9**-**14*), are particularly difficult to control because both are contagious among susceptible hosts. In contrast, bovine spongiform encephalopathy (BSE) does not appear to be contagious in cattle, but epidemics are sustained artificially through exposure to feed contaminated with infected bovine tissues (*15*); whether BSE in sheep is contagious remains undetermined (*16*). Both infected animals and environments apparently contaminated with the causative agent contribute to scrapie epidemics (*4**,**6**,**8*), and under some conditions, scrapie agents may persist in contaminated environments for years (*7*). Similarly, CWD is transmitted in the presence of infected mule deer (*O. hemionus*) (*10*), and circumstantial evidence exists for transmission from environments contaminated with the CWD agent (*9**,**11**,**14*). CWD epidemics do not appear to have been perpetuated by exposure to contaminated feed, but because ingestion of brain tissue can transmit CWD experimentally to deer (*11**,**17*), decomposed carcasses could serve as sources of infection in the environment.

Environmental sources of CWD infection represent potential obstacles to control in natural and captive settings. To investigate their role in transmission of this disease, we compared three potential sources of infection: infected live deer, decomposed infected deer carcasses, and an environment contaminated with residual excreta from infected deer.

## Materials and Methods

We conducted a replicated experiment to compare CWD transmission from three infection sources: naturally infected captive mule deer (one infected deer/paddock), carcasses from naturally infected captive mule deer that had decomposed in situ ≈1.8 years earlier (one carcass/paddock), or undisturbed paddock environments where infected mule deer had last resided 2.2 years earlier. Each exposure source was replicated in three separate paddocks; two clean paddocks served as unexposed controls. Control paddocks and paddocks where live infected deer were added or where carcasses decomposed were constructed specifically for this experiment; these paddocks had never housed captive deer or elk and had been closed to access by free-ranging cervids for ≈17 years. Because clinical courses varied in naturally infected deer that served as sources of direct exposure, actual exposure periods varied from 0.75 year (replicate 3) to 1 year (replicate 1). Excreta-contaminated paddocks previously held 19 mule deer that had been orally inoculated during a 2-year pathogenesis study (*11*) that ended 2.2 years before our study began (≈3.8 infected deer x years of excreta/paddock, assuming equal distribution) but that had not held deer or elk in the interim. All three carcasses were from mule deer euthanized in end-stage clinical CWD. They had been left to decompose in intact form except for the removal of small pieces of brainstem used to confirm CWD infection; only the skeletal remains of carcasses were present at the start of the study.

Experimental animals included 31 free-ranging mule deer from two donor populations distant to endemic CWD foci. Experimental animals were captured from the grounds of the Rocky Mountain Arsenal National Wildlife Refuge (n = 17) and the U.S. Air Force Academy (n = 14), Colorado. We assumed that all experimental animals were free from CWD when they were introduced into the experiment, and surveillance data provided evidence that deer obtained from these herds were uninfected before exposure. Surveillance for CWD in the source populations (*10**,**18*) showed 0 positive cases in a sample of 210 adult deer from the refuge and 0 positive cases in a sample of 65 adult deer from the academy.

We used these data to estimate the probability that infection could have been caused by transmission from animals from the source herds. To do so, we estimated one-sided, exact 99% binomial confidence intervals (BCI) on the proportion of each population that could be positive for CWD (refuge = 0–0.022, academy = 0–0.068). We then used the upper limit of this interval to estimate the maximum prevalence,_
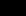
_, that could be reasonably expected in each of the source populations, given the inability to detect infections through surveillance. To assess whether observed results were likely due to preexisting infections, we treated each replicate (i.e., paddock) as an independent binomial experiment because the conditions in one paddock had no opportunity to influence the events in another paddock. Thus, for each replicate where infection occurred, we calculated the probability of at least one positive (i.e., "success") given the number of animals introduced to that replicate from the source population (i.e., "trials"), on the assumption that the probability of drawing a positive from the source population was _
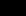
_. When two replicates within an exposure category showed infections, we estimated the probability that cases in both replicates resulted from introducing infected animals (and not from our experiment) as the product of the individual replicate probabilities.

We captured deer during March and May 2002 and transported them to the Colorado Division of Wildlife's Foothills Wildlife Research Facility, where they were confined in outdoor paddocks of ≈800 m^2^ (three replicate paddocks/exposure route, three deer/paddock); four deer were held in the two clean paddocks as unexposed controls. Each replicate of exposure paddocks was initially stocked with three mule deer. Shortly after arrival, one deer was moved to a different paddock within the same exposure condition to resolve social strife, and four fawns were born into three other paddocks; these changes are reflected in denominators in the Table. The distribution of prion protein genotype at codon 225 (serine [S]/phenylalanine [F] [19]) did not differ (Fisher exact test p = 0.6) among the four groups (three exposure groups + control).

Deer were fed alfalfa hay and a pelleted supplement; diets contained no animal protein or other animal byproducts. Individual paddocks and exposure blocks were physically segregated to prevent cross-transmission within and among exposure categories; dedicated clothing and equipment were used to minimize potential cross-contamination, but other potential fomites, like small mammals, birds, and insects, could not be controlled. However, transmission by routes such as these would be consistent with hypothesized transmission from environmental sources rather than direct animal-to-animal contact. After the animals had undergone ≈1 year of exposure to respective sources of infection, we obtained biopsied tonsil specimens from each participant deer and conducted an immunohistochemical analysis using anti-PrP MAb 99/97.6.1 (*20**,**21*). Upon detecting >1 infected deer in a paddock, we removed all inhabitants of that paddock and confirmed CWD infection in animals with positive biopsy results (*20*). Study protocols were reviewed and approved by the Colorado Division of Wildlife Animal Care and Use Committee.

## Results

Mule deer exposed to contaminated environments or to infected deer contracted CWD (Table). None of the unexposed deer were infected. One or more introduced deer became infected in two of three paddocks containing a naturally infected deer, in two of three paddocks containing a decomposed deer carcass, and in one of three paddocks contaminated with residual deer excreta (Table) within 1 year of exposure. Infected deer included unrelated animals from both donor herds (2/17, 3/14; Fisher exact test p = 0.64), as well as one of four fawns born during the study. Males (4/16) and females (2/15) were infected at equivalent rates (Fisher exact test p = 0.65); similarly, deer of all three codon 225 genotypes (SS = 6/26, SF = 0/7, FF = 0/2) were infected at equivalent rates (Fisher exact test p = 0.52). Deer with positive biopsy results appeared healthy and did not show signs of CWD, consistent with early (<1 year in duration) infections (*11**,**17*).

**Table:** Chronic wasting disease arising in mule deer exposed to environments contaminated by residual excreta, carcasses, or other infected deer

Replicate	Exposure source			
	Infected deer	Infected carcass	Residual excreta	Unexposed
1	1/4^a^	0/3	1/3	0/2
2	0/2	2/4	0/3	0/2
3	1/4	1/5	0/3	NA^b^
Total	2/10	3/12	1/9	0/4

^a^Number positive/number exposed (not including infected source deer). ^b^Not applicable; controls included only two replicate paddocks.

On the basis of prior data from surveillance of source populations, our results were not likely explained by the null hypothesis of infections introduced from the source populations (p = 0.036 for academy source deer and p < 0.0001 for refuge source deer). The probability of prior infection accounting for our results in the pattern observed (Table) was p < 0.0013 for the infected animal exposure, p < 0.037 for the carcass exposure, p < 0.064 for the excreta exposure, and overall p ≈ 0.000003 for the observed results arising from preexisting infections. Because these probabilities were based on one-sided, upper 99% BCIs, we can conservatively reject the null hypothesis of infection arising from the source populations. The only remaining possibility is that infections arose from experimental exposures that included environments harboring the infectious agent from excreta or decomposed carcasses.

## Discussion

Prions cannot be directly demonstrated in excreta or soil. However, CWD infection–specific protease-resistant prion protein (PrP^CWD^) accumulates in gut-associated lymphoid tissues (e.g., tonsils, Peyer patches, and mesenteric lymph nodes) of infected mule deer (*11**,**17**,**22*), which implicates alimentary shedding of the CWD agent in both feces and saliva (*10**,**11**,**17*). Because PrP^CWD^ becomes progressively abundant in nervous system and lymphoid tissues through the disease course (*11*), carcasses of deer succumbing to CWD also likely harbor considerable infectivity and thus serve as foci of infection. We could not determine the precise mechanism for CWD transmission in excreta-contaminated paddocks, but foraging and soil consumption seemed most plausible. Deer did not actively consume decomposed carcass remains, but they did forage in the immediate vicinity of carcass sites where a likely nutrient flush (*23*) produced lush vegetation (Figure).

**Figure:**
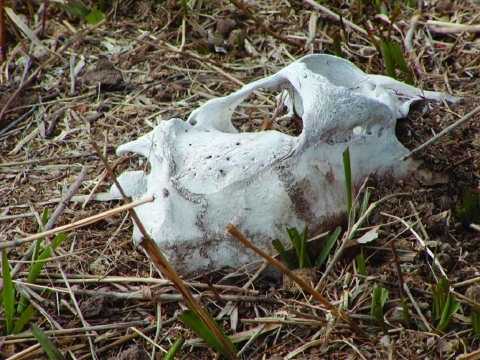
Green forage growing at the site where a deer carcass infected with chronic wasting disease had decomposed. Such sites were attractive to deer, as illustrated by the grass blades recently cropped in the experiment.

Our findings show that environmental sources of infectivity may contribute to CWD epidemics and illustrate the potential complexity of such epidemics in natural populations. The relative importance of different routes of infection from the environment cannot be discerned from our experiment, but each could play a role in sustaining natural epidemics. Although confinement likely exaggerated transmission probabilities, conditions simulated by this experiment do arise in the wild. Mule deer live in established home ranges and show strong fidelity to historic home ranges (*24**-**26*). As a result of such behavior, encounters with contaminated environments will occur more frequently than if deer movements were random. Feces and carcass remains are routinely encountered on native ranges, thus representing natural opportunities for exposure. Social behavior of deer, particularly their tendency to concentrate and become sedentary on their winter range, also may increase the probability of coming into contact with sources of infection in their environment.

The ability of the CWD agent to persist in contaminated environments for >2 years may further increase the probability of transmission and protract epidemic dynamics (*8*). Because infectivity in contaminated paddocks could not be measured, neither the initial levels nor degradation rate of the CWD agent in the environment was estimable. However, the observed persistence of the CWD agent was comparable to that of the scrapie agent, which persisted in paddocks for ≈1 to 3 years after removal of naturally infected sheep (*7*). Similarities between the CWD and scrapie agents suggest that environmental persistence may be a common trait of prions. Whether persistence of the BSE prion in contaminated feed production facilities or in environments where cattle reside contributed to BSE cases in the United Kingdom after feed bans were enacted (*27*) remains uncertain but merits further consideration.

Indirect transmission and environmental persistence of prions will complicate efforts to control CWD and perhaps other animal prion diseases. Historically, control strategies for animal prion diseases have focused on infected live animals as the primary source of infection. Although live deer and elk represent the most plausible mechanism for geographic spread of CWD, our data show that environmental sources could contribute to maintaining and prolonging local epidemics, even when all infected animals are eliminated. Moreover, the efficacy of various culling strategies as control measures depends in part on the rates at which the CWD agent is added to and lost from the environment. Consequently, these dynamics and their implications for disease management need to be more completely understood.
